# A Case Report on Renal Abscess: Rare Diagnosis in a Healthy Young Female With No Risk Factors

**DOI:** 10.7759/cureus.50069

**Published:** 2023-12-06

**Authors:** Kadijah Nyosha Chamba, Raymond Sheridan

**Affiliations:** 1 General Medicine, Royal Devon and Exeter Hospital, Exeter, GBR

**Keywords:** urological infection, complication of infection, intra-abdominal collection, collection, urinary tract infection, symptomatic uti, febrile uti, complicated uti, renal abscess drainage, renal abscess

## Abstract

Renal abscess is a rare complication of pyelonephritis known to be linked to recurrent urinary tract infections, anatomical abnormalities, obstruction, and the immunocompromised. Symptoms can be very nonspecific such as fever, chills, and abdominal or flank pain, which makes it more challenging to diagnose; however, this has been made easier with the help of medical imaging. Persistently spiking fevers despite antibiotics should prompt a review for possible abscess formation. We describe a case of renal abscess in a previously healthy young female with no risk factors who was successfully treated with antibiotics and radiologically guided percutaneous drainage.

## Introduction

A renal abscess is a collection of pus in the renal parenchyma. It is a rare complication of urological infections with very vague symptoms that cause patients to present as acutely unwell and septic. It can be caused by ascending infection or bacteria seeding from another septic focus from inside the body [1-3]. Certain risk factors such as anatomical abnormalities of the genitourinary tract, history of complicated or recurrent UTI, kidney stones and obstruction, immunodeficiency, pregnancy, and diabetes may predispose patients to form renal abscesses [1-3]. To prevent even further complications and reduce rates of mortality, it is crucial to diagnose a renal abscess early.

However, the challenge in diagnosing a renal abscess is that it frequently presents with nonspecific symptoms such as fever, chills, and abdominal or flank pain [1]. Previous complicated cases of renal abscess have been reported in [1-6], but most of these patients have at least one risk factor.

Most of the reported cases in the medical literature are in children or adults of ages over 60. Reported renal abscess cases in younger age groups such as in our patient's age group are lacking. In this case report, we describe the progress, diagnosis, and outcome of a renal abscess in a young female. This patient had no urogenital abnormality or any of the other aforementioned risk factors that contribute to a renal abscess, making this particular case a rare diagnosis.

## Case presentation

A 21-year-old fit and healthy female presented to our acute medical unit with a four-day history of left flank pain, fever, nausea, and dysuria associated with two days of vomiting. She had relocated to the United Kingdom from the United States of America four months ago for university. She had no history of urinary tract infection, renal stones, vesicoureteral reflux, or anatomical abnormalities of the genitourinary tract. She was not pregnant nor immunocompromised, had no other medical conditions, and was not sexually active.

On admission, she had low blood pressure and raised temperature (Table 1).

**Table 1:** Initial observations on admission

Observations	
Temperature	37.8 °C
Blood Pressure	91/64 mmHg
Heart Rate	82 bpm
Respiratory Rate	14 bpm
Oxygen Saturation (SpO2)	98% on room air

Her examination was unremarkable apart from mild left upper quadrant tenderness, and the highest recorded temperature on day one of admission was 39°C. Admission blood showed raised inflammatory markers, C-reactive protein (CRP) 196 mg/L, and white blood cells (WBC) 17.3 10*9/L with neutrophil predominance and slightly raised creatinine 89 umol/L. She was presumed to have a urinary tract infection and started on intravenous (IV) antibiotics as per hospital microbial guidelines, which were amoxicillin and gentamycin.

**Figure 1:**
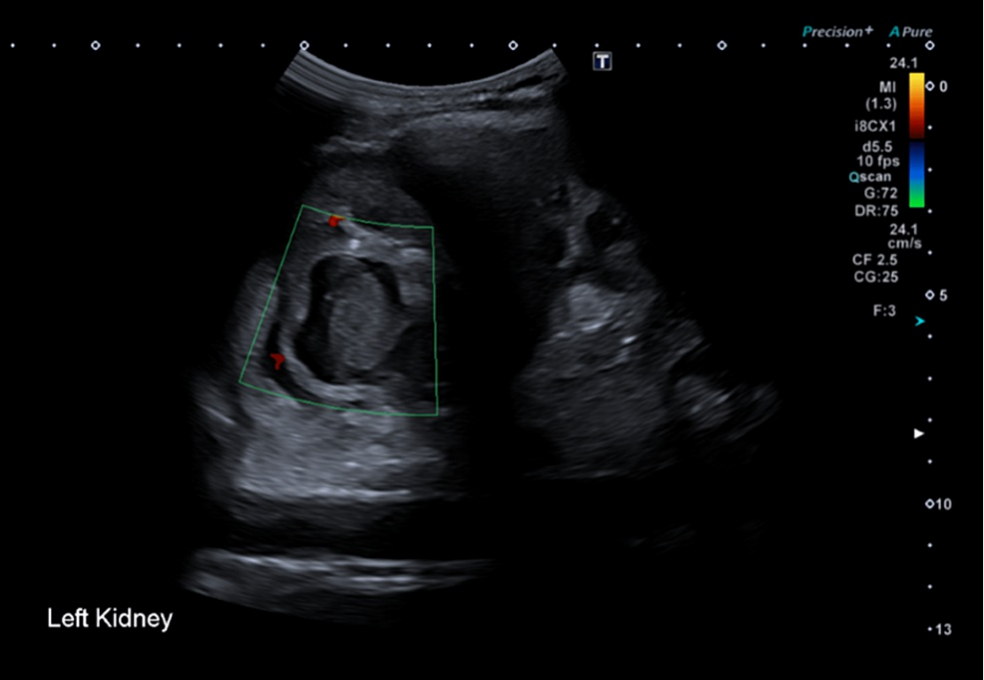
Ultrasound scan of the left kidney Expanded upper calyx in the left kidney containing a possible clot.

It was now four days into her admission, and she continued to spike temperatures daily despite being on IV antibiotics. Her blood and urine cultures showed no growth. This raised concerns about antibiotic resistance and other differentials. Microbiology was consulted, and a renal ultrasound scan (Figure 1) was requested. The scan reported “expanded upper calyx in the left kidney containing a possible clot, debris in the bladder and free fluid in the pelvis.”

**Figure 2:**
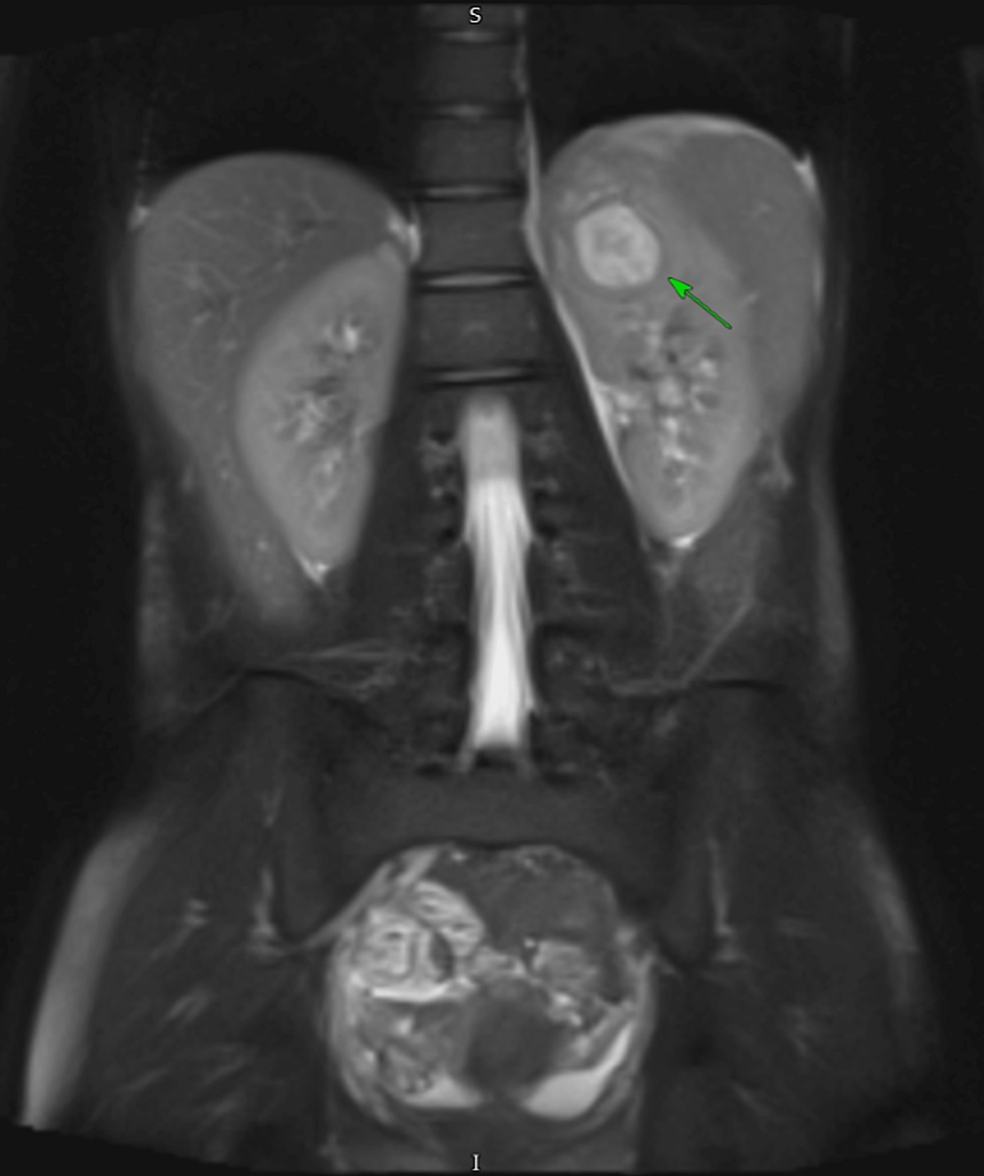
MRI of the left renal abscess

The ultrasound scan showed an unusual report of a possible clot, and she had ongoing temperature spikes even on day five despite antibiotics. We then went on to do an MRI pelvis (Figure 2), which confirmed a left renal abscess with reactive changes causing hepatosplenomegaly, bilateral pleural effusions, and a small volume of intraperitoneal fluid. It was thought that hepatosplenomegaly was likely because of her infection.

**Figure 3:**
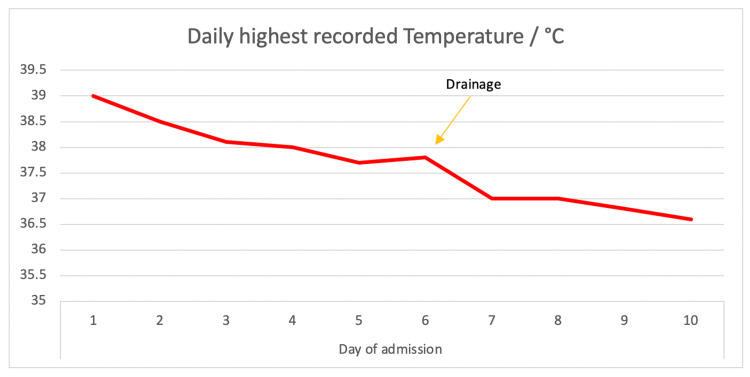
Temperature recorded during admission The arrow points to the drainage of the abscess.

**Figure 4:**
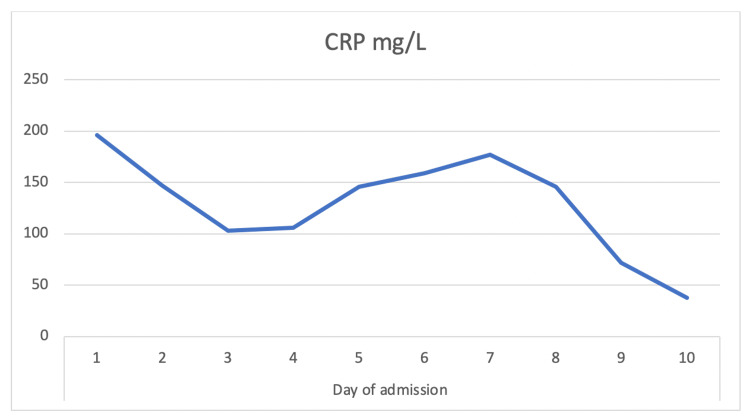
CRP trending down

An ultrasound-guided drain was placed into the left upper pole of a kidney with a pigtail (ureteric stent) on day 10 of her illness (day six of admission). A sample of the drained pus was sent and grew “gram-positive cocci in clusters.” It was methicillin-sensitive staphylococcus aureus (MSSA), which was sensitive to flucloxacillin. She had no fever spikes following drainage and her inflammatory markers came down significantly (Figures 3-4).

Her creatinine was back to normal, and she was discharged with a two-week course of oral flucloxacillin with a plan to repeat scans in two to three months to ensure that her liver and spleen had returned to their normal size. She has since recovered well.

## Discussion

The most common organisms that cause renal abscesses are E. coli, Proteus, and S. aureus [3]. Although pus drained from the abscess in this patient grew S. aureus (MSSA), we had no reason to suspect this particular organism. S. aureus is often found in bacteraemia and infective endocarditis. S. aureus infection sources are known to be through haematological spread or soft tissue and skin infections [7]. Our patient, however, had no skin lesions and had no other risk factors associated with this such as intravenous drug use, which is what makes this particular case so intriguing.

Diagnosing renal abscesses has been made easier because of more availability of medical imaging. Although it is not always able to detect them [4], an ultrasound is used most commonly and is a cost-effective, non-radiation exposure option for follow-up. A renal abscess on ultrasound would appear as a well-defined round structure with an echogenic centre (Figure 1). There is a discrepancy in detecting clots, and this prompted us to investigate further using an alternative imaging method.

Compared to an ultrasound, other tests such as computed tomography (CT) scans or magnetic resonance imaging (MRI) are far more accurate. These imaging methods are capable of showing even more detailed structures or configurations [4]. However, while CT scans are often recommended [4], they expose patients to high amounts of radiation. As our patient is a young female, we specifically chose an MRI to minimise her exposure to radiation. Nevertheless, ultrasound and CT scans have made diagnosing abscesses much easier and are also able to aid in treatment to guide drainage [4].

Undiagnosed and untreated renal abscesses can present with sepsis and may even result in death [1], so focus should be on early diagnosis and treatment. Renal abscesses can be successfully managed by longer durations of antibiotics or drainage [5]. According to EUA guidelines, drainage is required if the size is larger than 3 cm or if it does not resolve with antibiotics. Given that the patient may have recently travelled from another country as in our case, consideration needs to be given to the sensitivities of organism growth and microbial resistance patterns. As stated before even after five days of intravenous antibiotics, she was still spiking fevers; hence, a surgical option was contemplated. Radiologically guided drainage is minimally invasive and done under local anaesthetic causing the least damage to renal parenchyma [5]. Samples obtained from the abscess will then aid in growing organisms and microbial sensitivities. If percutaneous drainage is not successful, surgical drainage is considered. Drainage of the abscess coupled with antibiotics is the definitive management for abscesses and is tolerated well by patients.

## Conclusions

Renal abscesses, although rare, can present with symptoms that are very nonspecific. If patients are spiking fevers despite intravenous antibiotics - think abscess. While medical imaging has helped improve diagnosis, it is important to recognize what each imaging modality can or cannot tell us, for example, if an ultrasound scan reports a possible clot - think differentials, i.e., abscess. It is also imperative to understand your local antibiotic resistance patterns but be conscious of your patient’s likely resistance patterns.
